# ﻿Diversity of *Fusarium* associated banana wilt in northern Viet Nam

**DOI:** 10.3897/mycokeys.87.72941

**Published:** 2022-02-10

**Authors:** Loan Le Thi1, Arne Mertens*, Dang Toan Vu, Tuong Dang Vu, Pham Le Anh Minh, Huy Nguyen Duc, Sander de Backer, Rony Swennen, Filip Vandelook, Bart Panis, Mario Amalfi, Cony Decock, Sofia I.F. Gomes, Vincent S.F.T. Merckx, Steven B. Janssens

**Affiliations:** 1 Plant Resources Center, Hanoi, Vietnam; 2 Meise Botanic Garden, Nieuwelaan 38, BE-1860 Meise, Belgium; 3 IITA-Tanzania, c/o Nelson Mandela African Institution of Science and Technology, Duluti, Arusha, Tanzania; 4 Bioversity International, Willem de Croylaan 42, BE-3001 Leuven; 5 Naturalis Biodiversity Center, Leiden, The Netherlands; 6 Plant Ecology and Nature Conservation Group, Wageningen University, PO Box 47, NL-6700 AA Wageningen, The Netherlands; 7 Department of Evolutionary and Population Biology, Institute for Biodiversity and Ecosystem Dynamics, University of Amsterdam, Amsterdam, The Netherlands; 8 Department of Biology, KU Leuven, Belgium; 9 Department of Plant Pathology, Faculty of Agronomy, Vietnam National University of Agriculture (VNUA), Vietnam; 10 Laboratory of Tropical Crop Improvement, Department of Biosystems, KU Leuven; 11 Fédération Wallonie–Bruxelles, Service général de l’Enseignement universitaire et de la Recherche scientifique, Rue A. Lavallée 1, 1080 Bruxelles, Belgium; 12 Mycothèque de l’Université catholique de Louvain (MUCL, MBLA), Place Croix du Sud 3, B-1348 Louvain-la-Neuve, Belgium; 13 13Faculty of Biotechnology, Vietnam National University of Agriculture (VNUA)

**Keywords:** AAA Cavendish, ABB Tay banana, banana disease, *Foc*-Race 1, *Foc*-TR4, FOSC, fungal diversity, *Musalutea*, Viet Nam

## Abstract

*Fusarium* is one of the most important fungal genera of plant pathogens that affect the cultivation of a wide range of crops. Agricultural losses caused by Fusariumoxysporumf.sp.cubense (*Foc*) directly affect the income, subsistence, and nourishment of thousands of farmers worldwide. For Viet Nam, predictions on the impact of *Foc* for the future are dramatic, with an estimated loss in the banana production area of 8% within the next five years and up to 71% within the next 25 years. In the current study, we applied a combined morphological-molecular approach to assess the taxonomic identity and phylogenetic position of the different *Foc* isolates collected in northern Viet Nam. In addition, we aimed to estimate the proportion of the different *Fusarium* races infecting bananas in northern Viet Nam. The morphology of the isolates was investigated by growing the collected *Fusarium* isolates on four distinct nutritious media (PDA, SNA, CLA, and OMA). Molecular phylogenetic relationships were inferred by sequencing partial *rpb1*, *rpb2*, and *tef1a* genes and adding the obtained sequences into a phylogenetic framework. Molecular characterization shows that c. 74% of the *Fusarium* isolates obtained from infected banana pseudostem tissue belong to *F.tardichlamydosporum*. Compared to *F.tardichlamydosporum*, *F.odoratissimum* accounts for c.10% of the Fusarium wilt in northern Viet Nam, demonstrating that *Foc* TR4 is not yet a dominant strain in the region. *Fusariumcugenangense* – considered to cause Race 2 infections among bananas – is only found in c. 10% of the tissue material that was obtained from infected Vietnamese bananas. Additionally, one of the isolates cultured from diseased bananas was phylogenetically not positioned within the *F.oxysporum* species complex (FOSC), but in contrast, fell within the *Fusariumfujikuroi* species complex (FFSC). As a result, a possible new pathogen for bananas may have been found. Besides being present on several ABB ‘Tay banana’, *F.tardichlamydosporum* was also derived from infected tissue of a wild *Musalutea*, showing the importance of wild bananas as a possible sink for *Foc*.

## ﻿Introduction

For millions of people, bananas are an important food crop. With an annual global production of 153 million tons produced on 5.6 million hectares of land, a revenue of more than 26.5 billion Euro was generated in 2017 (FAO 2018). Particularly in Asia, Africa, Latin America, and the Caribbean, bananas support rural livelihood as most grown bananas are self-consumed or locally traded. As a result, any reduction in crop harvest directly affects the income, subsistence, and nourishment of thousands of smallholders.

One of the most important fungal plant pathogens impacting the cultivation of numerous agricultural crops is the ascomycete *Fusarium* (e.g., rice, coffee, tomato, melon, wheat; Dean et al. 2012). *Fusarium* has a considerable economic, social and biological impact on the daily livelihood of millions of people worldwide. Within the genus, *F.oxysporum* is one of the two most devastating pathogens, besides *F.gramineum*. The *F.oxysporum* species complex (FOSC) is responsible for wilt diseases of various crops (e.g., cotton and tomato wilt) but is mainly known from its massive impact on the banana industry (Panama disease). For more than 100 years, the fungus has affected banana production worldwide (Ploetz and Pegg 1997; Ploetz 2015a).

Nowadays, the worldwide banana export is still seriously affected by *Foc*, as most of its current production depends on the cultivation of members of the Cavendish subgroup (Buddenhagen 2009; Ploetz 2009). Although the triploid ‘AAA’ Cavendish cultivars were selected in the past century for their resistance against F.oxysporumf.sp.cubense Race 1 (*Foc*-Race 1), to which the initially grown Gros Michel cultivars were highly susceptible (Stover 1962a), Cavendish cultivars (e.g., Grand Naine, Williams) are highly susceptible to *Foc*-TR4. All *Foc* strains currently known (e.g., Race 1, Race 2, STR4, TR4) pose a huge threat for banana cultivation worldwide. Moreover, knowing that nearly half of the global banana production is derived from Cavendish clones and has become more popular for domestic use, a *Foc*-TR4 pandemic is still not averted to date (Ploetz 2015b; Zheng et al. 2018).

In the near future, *Foc* will further intensively spread in Asia, thereby significantly affecting important banana-producing countries such as China, the Philippines, Pakistan, and Viet Nam (Scheerer et al. 2018). For Viet Nam, the predictions are dramatic, estimating a loss in the banana production area for the country of 8% within the next five years and up to 71% within the next 25 years (Scheerer et al. 2018).

As a soil-borne fungus, *Foc* invades the root system from where it subsequently moves into the vascular tissue that gradually deteriorates. When reaching the corm, wilt occurs eventually, resulting in the death of the contaminated plant (Stover 1962a). A particular problem that arises with *Foc* infections is the remaining presence of *Foc* spores (microconidia, macroconidia, and chlamydospores) in the soil surrounding the infected plants for at least 20 years after the complete removal of all infected plants or plant tissue (Stover 1962b; Buddenhagen 2009; Dita et al. 2018). As a result, reinfection of new banana accessions in the same area is very likely to happen in the absence of complete soil disinfection or if one has not waited long enough for planting new *Musa* cultivars (Moore et al. 2001; Huang et al. 2012). Therefore, Fusarium wilt not only has an impact on the overall yield during the time of infection but also on the land use for banana cultivars during the coming 20 years.

Whereas pathogenic *Foc* lineages were usually classified into three races (*Foc* 1, 2 & 4) based on the different *Musa* cultivars they had infected, the development of the Vegetative Compatibility Group (VCG) system resulted in a more in-depth identification tool of *Foc* strains into 24 unique entities (Fourie et al. 2011; O’Donnell et al. 2009; Perez-Vicente et al. 2014; Mostert et al. 2017). The fact that isolated *Foc* lineages could already be split up into compatible vegetative groups already indicated that there are more natural lineages in the FOSC than can be reflected by the number of races. In addition, the polyphyletic nature of F.oxysporumf.sp.cubense isolates is also demonstrated by Maryani et al. (2019a), who used a combined molecular phylogenetic approach to delineate natural lineages within the FOSC (O’Donnell and Cigelnik 1997), thereby describing 11 new *Fusarium* species which were formerly considered as *F.oxysporum*. A result of Maryani et al. (2019a) also indicated that the VCG system is perhaps slightly prone to an oversimplification of the categorization of different *Foc* strains that cause Fusarium wilt in bananas and plantains.

In 1968, Vakili and coworkers published the first survey on *Fusarium* infecting bananas in Southern Viet Nam (Vakili et al. 1968). Later studies showed that by the end of the 20^th^ century, *Foc* infections were omnipresent in the whole country (Mai Van Tri 1997; Bentley et al. 1998; Vinh et al. 2001). The characterization of the *Fusarium* isolates in the studies mentioned above demonstrated that Fusarium wilt on bananas in Viet Nam was derived from different *Foc*VCG’s (e.g., VCG 0123, VCG 0124, VCG 0124/5, VCG 0125). Hung et al. (2017) reported the first observation of *Foc*-TR4 (VCG 01213/16) on Cavendish bananas in Viet Nam using a combined molecular (polymerase chain reaction (PCR) approach) and morphological characterization. However, Zheng et al. (2018) claimed that they made the earliest collected records of *Foc*-TR4 in Viet Nam in 2016 by assessing the pathogenicity of the collected strains and characterizing them molecularly using whole-genome sequencing methodology. The study of Mostert et al. (2017) also used a molecular-morphological characterization approach to determine the origin of the different *Fusarium* infections in Viet Nam. Their results showed the presence of at least five different VCG’s (VCG 0123, VCG 0124, VCG 0124/5, VCG 0128, VCG 01221), of which the latter two were not yet detected in earlier studies.

In the current study, we aim to assess the overall diversity of *Foc* wilt in northern Viet Nam by using a combined morphological-molecular phylogenetic approach in which the different VCG’s are included. With this approach, we provide the overall species identity and phylogenetic position of *Foc* infections in the northern Vietnamese region and examine the genetic diversity between the different *Foc* isolates (from wild and cultivated bananas) collected from various provinces in northern Viet Nam. Furthermore, our results will indicate the proportion of the different *Foc* strains (and linked VCGs) that are currently infecting bananas in northern Viet Nam.

## ﻿Material and methods

### ﻿Sampling

**Figure 1.**
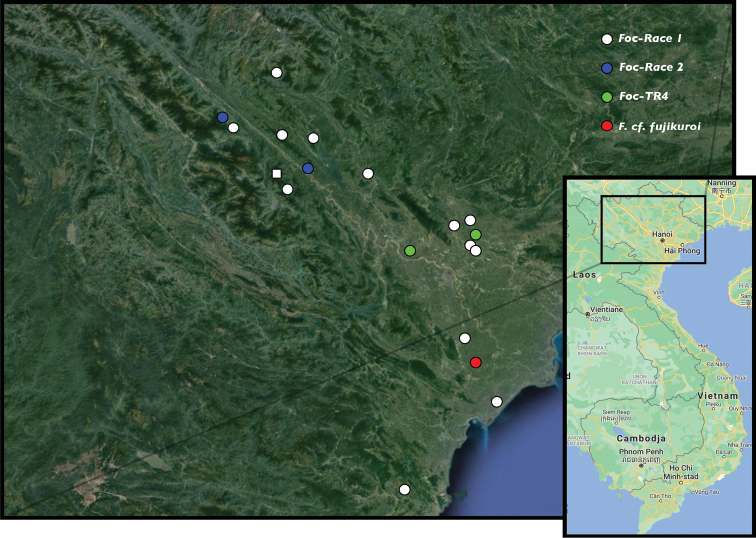
Distribution map of localities in northern Viet Nam where Fusarium wilt was observed. Colours indicate different *Fusarium* strains or species. Squares indicate *Fusarium* infections of wild bananas; circles indicate infections of cultivated bananas.

**Figure 2.**
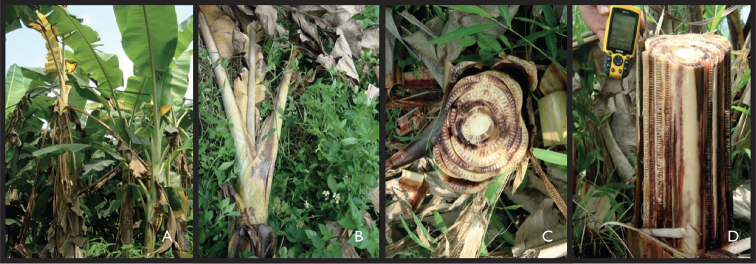
**A** overall view of a banana plant infected by *Fusarium* wilt **B** detailed view of wilted plant **C** radial cutting of *Fusarium*-infected banana pseudostem **D** tangential cutting of *Fusarium*-infected banana pseudostem.

From April 2018 until December 2019, several field trips were carried out focusing on the presence of banana Fusarium wilt in northern Viet Nam. During these surveys, banana Fusarium wilt samples were collected at 19 locations in three large geographic regions; North-eastern region, North Central region and Red River Delta, with most specimens collected in the latter region (Table 1, Fig. 1). *Fusarium* infected banana plants were identified by following a set of diagnostic characters in which (mostly older) leaves were clearly yellow (initiated from the leaf margin) or even completely collapsed, halfway along the petiole forming a ring of dead leaves around a dying plant, combined with brown discoloration and longitudinally fissuring of the pseudostems leaf sheaths (Fig. 2). From symptomatic plants observed in the field, discoloured brownish vascular tissue was collected from pseudostems and roots. Subsequent to the collection, infected tissue samples were stored in paper bags and put in a refrigerator or cooling box to avoid quality loss upon the further analysis in the molecular lab. For each sample collected, notes were taken about the altitude, longitude and latitude, site location, and the host specimen. Collected *Fusarium* samples were stored at the Plant Resources Center (PRC), Ha Noi, Viet Nam (Table 1).

**Table 1.** List of collected Fusarium (Foc) wilt samples on bananas in northern Viet Nam.

Isolate	Locality	Cultivar or Species	Altitude (m)	Latitude	Longitude
FOC1	Yen Binh town, Quang Binh district, Ha Giang province	Tay banana (ABB)	78	22°23'5.28", 104°32'54.0"	
FOC2	Dong Cay village, Yen Thang commune, Luc Yen district, Yen Bai province	Tay banana (ABB)	188	22°05'46.2", 104°45'34.0"	
FOC4	Khe Chao village, Ngoi A commune, Van Yen district, Yen Bai province	Tay banana (ABB)	76	21°54'21.4", 104°43'48.3"	
FOC5	No 7 village, Dai Son commune, Van Yen district, Yen Bai province	*Musalutea*	351	21°48'17.6", 104°36'01.0"	
FOC6-1	No 4 village, Dai Son commune, Van Yen district, Yen Bai province	Tay banana (ABB)	352	21°48'16.9", 104°36'02.5"	
FOC7	No 18 village, Lam Giang commune, Van Yen district, Yen Bai province	Tay banana (ABB)	137	22°02'35.3", 104°30'52.3"	
FOC10	Hai Son 1 village, Phu Nhuan commune, Bao Thang district, Lao Cai province	Tay banana (ABB)	370	22°13'58.6", 104°08'14.9"	
FOC11	Khanh Yen town, Van Ban district, Lao Cai province	Tay banana (ABB)	383	22°06'53.9", 104°14'37.9"	
FOC16	Hoang An commune, Hiep Hoa district, Bac Giang province	Tay banana (ABB)	20	21°23'00.2", 105°58'40.5"	
FOC18	Hoang Thanh commune, Hiep Hoa district, Bac Giang provinse	Tay banana (ABB)	18	21°23'22.1", 106°00'24.9"	
FOC21	Thinh Long town, Hai Hau district, Nam Dinh province	Tay banana (ABB)	4	20°01'59", 106°12'49.0"	
FOC23-2	Thanh Chau commune, Phu Ly district, Ha Nam province	Tay banana (ABB)	7	20°31'24.2", 105°55'31.1"	
FOC24	Hung Thanh commune, Tuyen Quang city, Tuyen Quang province	Tay banana (ABB)	26	21°48'19.4", 105°11'39.7"	
FOC25-1	Le Chi commune, Gia Lam district, Ha Noi province	Tay banana (ABB)	11	21°18'20.5", 106°00'24.6"	
FOC25-2	Le Chi commune, Gia Lam district, Ha Noi province	Tay banana (ABB)	11	21°18'20.5", 106°00'24.6"	
FOC38	Quan Mia village, Nghia Tan commune, Nghia Dan district, Nghe An province	Tay banana (ABB)	85	19°19'07.5", 105°21'53.7"	
FOC56	Agriculture University,Trau Quy town, Gia Lam district, Ha Noi province	Tay banana (ABB)	11	21°18'20.5", 106°00'24.6"	
FOC58	Nui Ngam, Minh Tan commune, Vu Ban district, Nam Dinh province	Tay banana (ABB)	3	20°21'59.1"N, 106°04'02.9"E	
FOC 61	Hong Chau commune, Yen Lac district, Vinh Phuc province	Cavendish (AAA)	3	21°10'17.1"N, 105°34'37.0"E	

### ﻿Isolate cultivation

In order to observe possible morphological differences between the Fusarium wilt isolates collected from the wild and cultivated northern Vietnamese *Musa* accessions, we followed the approach of Groenewald et al. (2006) in which different *Foc* isolates were grown on different media. Infected discoloured pseudostem tissue samples were cut into 2–3 cm pieces and placed on the Komada medium (Komada 1975). After a few days, fungal *Fusarium* colonies were transferred to plates with different media and then put in a growing chamber at 25 °C until the colonies reached 2–3 cm. The different isolates were grown on four distinct nutritious media to observe the Fusarium wilt in different culture conditions: PDA (Potato Dextrose Agar), SNA (Spezieller Nährstoffarmer Agar), CLA (Carnation Leaf Agar), and OMA (Oatmeal Agar) (Nirenberg 1976). The PDA medium consisted of 200g potato dextrose, 20g D-glucose, and 20g agar dissolved in 1000ml distilled water, whereas the SNA medium consisted of 1g KH_2_PO_4_, 1g KNO_3_, 0.5g MgSO_4_•7H_2_O, 0.5g KCl, 0.2g D-glucose and 0.2g D-sucrose dissolved in 1000ml distilled water. The CLA medium contained aseptic carnation leaves and 20g agar dissolved in 1000ml distilled water. The OMA medium consisted of 50g oatmeal and 20g agar dissolved in 1000ml distilled water. The growth of the *Fusarium* isolates on the different media took place under in-vitro conditions with temperatures between 23 and 27 °C (Pérez et al. 2003). After seven days of incubation, the developing colonies were morphologically investigated under a light microscope (400x magnification). The coloration of the colony, the morphology, and the size of the conidia were determined. The colony reverse colour was determined on PDA medium after incubation at room temperature, using the colour charts of Rayner (1970). In addition to colony colour, the aroma of the different cultures was assessed as a strong rank odour generated by mature cultures which is a typical characteristic for TR4 infections. In the first stage of culturing, we characterized the isolates as *Fusarium* spp. emanated from mycelium morphology and the presence of different types of conidia. The study of Maryani et al. (2019a) was used as a reference to classify the Foc lineages into different sublineages further. All obtained *Fusarium* isolates were stored in the Plant Resources Center (PRC), Ha Noi, Viet Nam.

### ﻿Molecular protocols

In order to extract high-quality DNA from the Fusarium wilt isolates collected and cultured, we used the pure mycelium cultures generated for the morphological characterization of the banana wilt. Total genomic DNA was isolated using a modified CTAB protocol based on the study of Lin et al. (2008) and Dellaporte et al. (1983). After the addition of 5ml TNE buffer (100 mM Tris-HCl, 50 mM EDTA, 50 mM NaCl, 8 μM β-mercaptoethanol, 1% SDS, pH 8.0) to the sampled mycelium, the samples were incubated for 1 h (65 °C). Subsequent to the lysis phase, 1.66ml NaOAc (5M) was added and centrifuged. Chloroform-isoamylalcohol (24/1 v/v) extraction was done twice, followed by isopropanol precipitation at -32 °C for 12h. After centrifugation at 4 °C, the pellet was washed twice (75% ethanol), air-dried, and dissolved in 100µl TE buffer (10mM TrisHCl, 0.1mM EDTA; pH 8)

Amplification reactions of *rpb1*, *rpb2*, and *tef1a* were carried out using standard PCR (20µl). Reactions were initiated with a 3 min heating at 95 °C followed by 30 cycles consisting of 95 °C for 30s, 55–65 °C (*rpb1* and *rpb2*) and 53°-59 °C (*tef1a*) for 60s, and 72 °C for 60s. Reactions ended with a 3 min incubation at 72 °C. Primers designed by O’Donnell et al. (1998) were used to sequence *tef1a*, whereas primers for *rpb1* and *rpb2* were adopted from O’Donnell et al. (2010). PCR products were purified using an ExoSap purification protocol. Purified amplification products were sequenced by the Macrogen sequencing facilities (Macrogen, Seoul, South Korea).

### ﻿Phylogenetic analyses

Raw sequences were assembled using Geneious Prime (Biomatters, New Zealand). Automatic alignment was conducted with MAFFT (Katoh et al. 2002) using an E-INS-i algorithm, a 100PAM/k = 2 scoring matrix, a gap open penalty of 1.3, and an offset value of 0.123. Manual fine-tuning of the aligned dataset was performed in Geneious Prime.

*Fusarium* sequence data of *rpb1*, *rpb2*, and *tef1a* was extracted from GenBank (September 20, 2020) using the ‘NCBI Nucleotide extraction’ tool in Geneious Prime. Together with the newly generated sequences for the 19 Vietnamese Fusarium wilt accessions, the total sequence data matrix consisted of 529 specimens divided over 201 species (Suppl. material 1: Table S1). Of those, 72 species belong to different closely related genera of *Fusarium* within the Nectriaceae family were chosen as outgroup (*Albonectria*, *Bisifusarium*, *Cosmospora*, *Cyanonectria*, *Cylindrocarpon*, *Fusicolla*, *Geejayessia*, *Luteonectria*, *Macroconia*, *Microcera*, *Neocosmospora*, *Pycnofusarium*, *Rectifusarium*, *Setofusarium*; sensu Crous et al. 2021). Newly generated sequences were deposited in the GenBank sequence database (Table 1). Furthermore, in order to compare the newly collected Vietnamese *Foc* accessions with the known VCGs, the sequence dataset included *Foc* samples representing all VCGs (see Table S1; Ordonez et al. 2015), except for VCG01212 and VCG0129, from which only one locus was available, thereby causing phylogenetic biases due to the occurrence of too much missing data.

Possible incongruency between the different datasets was inferred by conducting an ILD test (Farris et al. 1995) as implemented in PAUP* v.4.0b10 (Swofford 2003) with the following parameters applied: simple taxon addition, TBR branch swapping, and heuristic searches of 1000 repartitions of the data. Despite the well-known sensitivity of the ILD test (Barker and Lutzoni 2002), the results of this test were compared in light of the resolution and support values for each of the single gene topologies. As a result, the possible conflict between data matrices was visually inspected by searching for conflicting relationships within each topology (obtained per single sequence data matrix) that were supported by a Maximum Likelihood (ML) support value > 70% (hard vs. soft incongruence; Johnson and Soltis 1998; Pirie 2015). A conflict was assumed to be significant if two different relationships for the same set of taxa (one being monophyletic and the other non-monophyletic) were observed in rival trees. ML analyses were conducted under the RAxML search algorithm (Stamatakis 2014) with the GTRGAMMAI approximation of rate heterogeneity for each gene. ML bootstrapping was carried out on five hundred bootstrapped datasets using the RAxML Rapid bootstrap algorithm (ML-BS).

The best-fit nucleotide substitution model for each dataset was selected using jModelTest 2.1.4. (Posada 2008) out of 88 possible models under the Akaike information criterion (AIC). The GTR+I+G, TVM+G, and HKY+I+G were determined as the best-fit substitution models for *rpb1*, *tef1a*, and *rpb2* markers, respectively. Consequently, we used a mixed-model approach to apply different evolutionary models on each DNA region of the combined dataset (Ronquist and Huelsenbeck 2003). Bayesian inference analyses were conducted with MrBayes v3.2.6 (Ronquist et al. 2012) on three individual data partitions and a combined data matrix. Each analysis was run twice for 20 million generations. Trees were sampled every 5000^th^ generation. Chain convergence and ESS parameters were inspected with TRACER v.1.4 (Rambaut and Drummond 2007). Only nodes with Bayesian posterior probabilities (BPP) above 0.95 were considered as well supported by the data (Suzuki et al. 2002).

## ﻿Results

Fusarium wilt infections are prevalent in most of northern Viet Nam as they have been observed in all provinces of northern Viet Nam that were sampled in this study. The 19 Fusarium wilt infections collected based on the typical plant Fusariosis symptoms (old leaves turning yellow, leaves gradually collapsing, petioles broken close to the midrib with dead leaves remaining attached to the pseudostem, pseudostem sheaths longitudinally splitting near the base, and vascular necrosis) (Fig. 2) were cultured and further morphologically and molecularly analysed. Despite the fact that tissue which showed signs of Fusariosis disease symptoms was used to further morphologically and molecularly characterize the *Fusarium* wilt, we cannot draw eminent conclusions on the pathogenicity of the isolates as no real pathogenicity test was carried out and therefore Koch’s postulates were not fully fulfilled.

Morphological characterization of the cultured pathogenic Fusarium wilt isolates showed that when the isolates were grown on CLA medium, they produced macroconidia that were uniform in size and form. On SNA medium, the morphology of the macroconidia was sometimes less uniform in size than when SLA medium was used. Except for two accessions (FOC56 and FOC61), no aroma was observed among the *Fusarium* isolates collected in northern Viet Nam. In general, for all isolates, we observed that macroconidia are sickle-shaped, 3–7 septate, and thin-walled. Microconidia are oval to kidney-shaped, 0–1 septate. Chlamydospores were round and thick-walled. Subtle differences have been observed in the colony morphology and coloration. Based on these morphological differences, we tried to identify different groups within the *Fusarium* isolates analysed.

The first group, consisting of 14 isolates (FOC1, 2, 5, 6–1, 7, 11, 16, 18, 21, 23–2, 24, 25–1, 25–2, 38), is characterized by a purple reverse in the centre, white-greyish towards the periphery. The colony surface is dry and is filamentous at the edge. On CLA medium, it produces ample macroconidia, yet only little microconidia. On PDA and SNA medium, it produces prolific microconidia. The second group has a reverse colony colour containing a small touch of dark purple in the centre, gradually discolouring to white towards the edge. This type is observed for isolates FOC 4 and 10. The surface of these colonies is also dry and filamentous at the margin. On CLA medium, ample macroconidia are produced, whereas on PDA and SNA medium, the presence of macroconidia is less profound. On the latter two media, prolific microconidia are produced. The third group of isolates (FOC56 and 61) is characterized by an unpigmented, white colony reverse and a dry colony surface with a filamentous margin. On CLA medium, many macroconidia are produced, while on PDA and SNA medium, macroconidia are hardly formed. On PDA and SNA, prolific microconidia are produced, whereas on CLA medium, only a few microconidia were observed. In addition, FOC 56 and 61 isolates are characterized by a typical strong odour of the older cultures. FOC 58 falls a bit amidst the first and second group, containing a pale purple colony reverse colour that becomes whitish towards the periphery and with a dry colony surface appearance.

### ﻿Phylogenetic analyses of *Fusarium* wilt isolates from northern Viet Nam

Sequence characteristics of all data matrices analysed are summarized in Table 2. Despite the fact that sometimes not all gene markers could be sequenced, their absence did not influence the overall phylogenetic results, as sufficient nucleotide variation was present. The partition homogeneity test found no significant incongruence between all three sequence datasets (with all P-values being larger than 0.05). Visual examination of the two different partitions of the combined dataset corroborates this congruency analysis.

**Table 2.** Alignment and sequence characteristics of the different partitions (including outgroup specimens).

	*rpb1*	*rpb2*	*tef1a*
N° taxa	457	525	271
Sequence length range	558–1574	597–859	343–636
Aligned sequence range	1578	917	797
Variable characters	1103 (70%)	572 (62%)	529 (66%)
Constant characters	474	345	268

**Figure 3.**
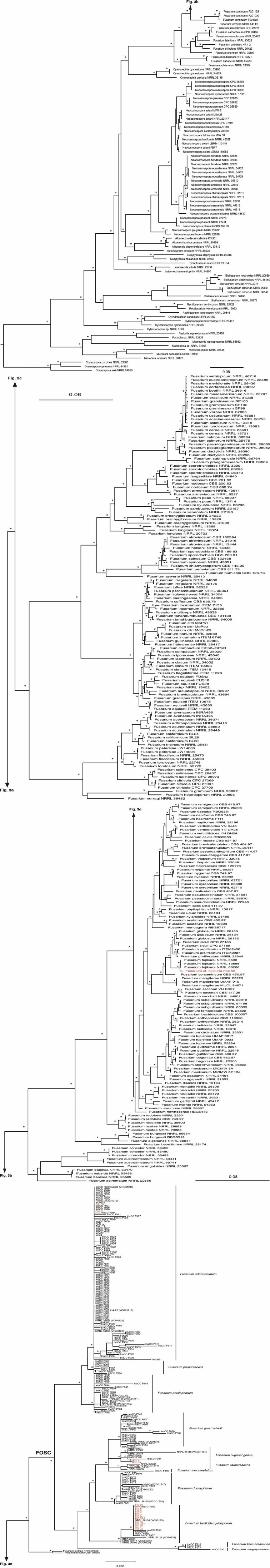
Maximum Likelihood topology obtained via heuristic search algorithm of the combined *rpb1*, *rpb2* and *tef1a* data matrix. Bootstrap support (ML-BS) values above 50 are indicated with a dot, ML-BS values above 75 are indicated with an asterisk. No indication above the branches indicates a ML-BS value below 50. Newly included accessions are indicated in red. FOSC: *Fusariumoxysporum* species complex, FFSC: *Fusariumfujikuroi* species complex.

Phylogenetic analyses of the 19 Fusarium wilt isolates found in various northern Vietnamese bananas showed that although overall morphological characterisation pointed towards F.oxysporumf.sp.cubense, it was clear that they had various evolutionary origins (Fig. 3c, d). Of the 19 accessions analysed, two (FOC61 and FOC56) were placed within the *F.odoratissimum* clade (as defined by Maryani et al. 2019a, Fig. 3d) and for which pathogenicity tests by Maryani et al. (2019a) showed that the members of this group caused infections in Cavendish and Gros Michel AAA banana varieties. In addition, VCG 01213 and VCG 01216 are positioned close to FOC61 and FOC56. As a result, two of the 19 (10.5%) northern Vietnamese Fusarium wilt isolates are assumed to be *Foc*-TR4 (also taking the morphological characterization into account). Interestingly, one of the two isolates characterised as *Foc*-TR4 (FOC61) infected a Cavendish plantation in Vinh Phuc province, whereas the other infection of *Foc*-TR4 (FOC56) took place on ABB Tay banana cultivars situated on a smallholder farm in Nam Dinh province (Table 1). The largest group (13 accessions; 68.5%) of Fusarium wilt isolates in northern Vietnamese bananas belong to the recently delineated *F.tardichlamydosporum* clade (Fig. 3d). Pathogenicity tests carried out for this clade by Maryani et al. (2019a) have indicated a large infection rate in Gros Michel cultivars for this lineage, and therefore members of the *F.tardichlamydosporum* clade are consequently classified as *Foc*-Race 1. Furthermore, the isolates that fell within the *F.tardichlamydosporum* were also most closely related to VCG 0125, a known *Foc*-Race 1 representative. In northern Viet Nam, infections of *Foc*-Race 1 occurred both on wild and cultivated accessions. For the cultivated accessions, the *Foc*-Race 1 was only found on the ABB Tay banana cultivar, yet was clearly spread in northern Viet Nam as it was found in eight different provinces (Ha Giang, Yen Bai, Lao Cai, Bac Giang, Nam Dinh, Ha Nam, Tuyen Quang, Ha Noi; Table 1). Most interestingly, *Foc*-Race 1 was also identified (isolate FOC5) in an individual of the wild banana *Musalutea* (section Callimusa). Here the infected accession grew sympatrically with other individuals of *M.lutea* as well as with *M.itinerans*. The area where this infection occurred was a steep, abandoned rice terrace in Yen Bai province where hundreds of individuals of both wild species co-occurred and were rather close to one of the smallholder farms where *Foc*-Race 1 was also detected (isolate FOC6-1). In addition to the *Foc*-Race 1 infections caused by *Fusariumtardichlamydosporum*, *F.duoseptatum* is also classified as a *Foc*-Race 1 Fusarium wilt (see Maryani et al. 2019a). An infection of this latter *Foc* isolate (FOC38) was found only once in northern Viet Nam (Nghe An province; c. 5% of the Fusarium wilt infections), where it infected the ABB Tay banana cultivars that were grown on a smallholder farm. The VCGs that occurred in the same clade as FOC38 are VCG 01223 and VCG 01217, with the latter being known as a *Foc*-Race 1 representative (e.g., Katan 1999; Fraser-Smith et al. 2014).

In addition to the *Foc*-Race 1 and *Foc*-TR4 infections, two *Foc* isolates (FOC4 and FOC10) were found in northern Viet Nam (10.5%) that belong to the recently described *F.cugenangense* (Maryani et al. 2019a; Fig. 3d). Hitherto, this *Fusarium* species was considered to be strictly Indonesian (see Maryani et al. 2019a). Pathogenicity tests conducted for representatives of *F.cugenangense* by Maryani et al. (2019a) have demonstrated that it only causes a mild infection in Gros Michel and Cavendish and is regarded as non-pathogenic for the above-mentioned AAA cultivars. However, our results clearly show that the infection of this isolate also occurred on ABB Tay banana cultivars in northern Viet Nam, where it had a large impact on the fitness of the infected host plants. Although additional confirmation is needed, Maryani et al. (2019a) assume that representatives of the *F.cugenangense* clade should be considered as *Foc*-Race 2 (Maryani et al. 2019a; Fig. 3d), yet more thorough analyses need to be carried out in order to further confirm this hypothesis. The VCG that occurred in the same clade as FOC4 and FOC10 is VCG 01221.

A final Fusarium wilt infection (FOC58) that was regarded upon collection in the field and during morphological screening as a *Foc* infection (c. 5% of the Fusarium wilt infections observed in this study) was not situated in the *F.oxysporum species complex* (FOSC) but was a distinct lineage sister to *F.fujikuroi* (Fig. 3c), a well-known pathogen of rice (e.g., Wulff et al. 2010; Choi et al. 2018). This *Fusarium* isolate was the prime infection source of ABB Tay bananas cultivated on a small plantation for local use in Nam Dinh province (Fig. 1).

## ﻿Discussion

### ﻿*Fusarium* wilt in Vietnam: lineage identification

To better manage the significant threat of *Foc* dispersion in northern Viet Nam, the correct identification and abundance of the *Foc* strains that cause Fusarium wilt in bananas in the region are necessary. This is the basis for eradication-confinement and suppression-contention measures (Perez-Vicente et al. 2014). Since the survey of Vakili et al. (1968), *Foc* Race 1 has been considered as the main *Foc* infecting edible bananas in Viet Nam. With the emergence of *Foc* TR4 it remained unclear how abundant this new pathogenic *Foc* strain had become in Viet Nam. Although officially present in Viet Nam for only a few years (Hung et al. 2017; Zheng et al. 2018), *Foc* TR4 was already observed on Cavendish bananas in 1998 in Southern China (Hu et al. 2006). A few years later, in 2002, *Foc* TR4 was also found in Chinese regions adjacent to northern Viet Nam (Hu et al. 2006; Li et al. 2013). With the current shift of Cavendish cultivation in Asia from China to its neighbouring countries Laos, Myanmar, and Viet Nam, there is also an active spread of *Fusarium* pathogens through transportation of planting material, farming equipment, and contaminated soil from China (Zheng et al. 2018), so that *Foc* TR4 can quickly become the most dominant *Foc* race in Viet Nam affecting banana cultivation.

The present study applies the FOSC species delimitation concept of Maryani et al. (2019) to delineate the *Foc* lineages sampled in northern Viet Nam more thoroughly. Furthermore, incorporating the different VCGs in the current phylogenetic dataset allowed us to link the Vietnamese *Foc* isolates with one of the currently known VCGs that have been assessed in the past. Based on the compatibility of the novel material with the VCGs present in the same clade and their specific species allocation following the species delineation concept of Maryani et al. (2019), we linked the northern Vietnamese *Foc* isolates to one of the known *Foc* Races. Accordingly, our results show that *Foc* isolates that are phylogenetically situated within a lineage containing *Foc* Race 1 are among the most common isolates in northern Viet Nam, causing 74% of all the infections. A more in-depth molecular characterisation shows that of these, 13 out of 14 isolates are representatives of *F.tardichlamydosporum*, whereas one isolate is a representative of *F.duoseptatum*. Whereas *F.tardichlamydosporum* is commonly present throughout the northernly oriented Northeastern region and Red River Delta, *F.duoseptatum* is not present in these more northerly oriented geographic regions but occurs in the more centrally oriented North Central region in Viet Nam. Also, from a global distributional perspective, *F.tardichlamydosporum* is much more widespread than *F.duoseptatum*, with the first species located in Australia, Indonesia, Malaysia, Honduras, and Brazil, and the latter is only known to date from Indonesia and Malaysia (Maryani et al. 2019a).

Interestingly, the *Foc* isolates that are phylogenetically situated within the *F.odoratissimum* species lineage (linked to *Foc*-TR4 infections; Maryani et al. 2019a) account for only 10% of the Fusarium wilt in northern Viet Nam, demonstrating that *Foc*-*TR4* has not yet become a dominant banana pathogen, unlike other countries in Asia where there is a tendency to grow Cavendish cultivars as large monocultures, such as in China, the Philippines, and Taiwan. The *F.odoratissimum* accessions found in the current study were located in the River Delta region of northern Vietnam, provinces Vinh Phuc and Nam Dinh, which are rather distant from *Foc*-TR4 infected regions in Southern China. This indicates a gradual spreading in Viet Nam of *F.odoratissimum* (=F.oxysporumf.sp.cubense TR4) towards the south as the TR4 isolates analysed by Zheng et al. (2018) were collected in the upper North of Viet Nam in the Lao Cai province at only a few kilometres from the border with China (Yunnan). At the moment, it is unclear whether the occurrence of *Foc*-TR4 in Viet Nam is still in an initial lag phase, with the potential of largely increasing its distribution range in the country if conditions would enable the disease to spread (Pegg et al. 2019). Especially the replacement of citrus plantations and maize fields by Cavendish monocultures provides an ideal basis for *Foc*-TR4 to rapidly spread as plants available for infection become less limited. A more worrying observation is that *Foc*-TR4 is not only found in Cavendish bananas in Viet Nam, but it also poses a threat to local banana varieties as is observed in the current study where *F.odoratissimum* is found in ABB Tay banana cultivars.

The current study demonstrates that the presence of *F.cugenangense* (linked to *Foc*-Race 2 infections; Maryani et al. 2019a) also accounts for 10% of the Fusarium wilt in northern Viet Nam. In general, *Foc*-Race 2 infections occur on triploid ABB Bluggoe varieties and its closely related cooking cultivars (Jones, 2000). Besides Bluggoe cooking bananas, *Foc*-Race 2 also infects the tetraploid AAAA Bodles Altafort hybrid between Gros Michel (AAA) and Pisang Lilin (AA) (Stover and Simmonds 1987). In addition, experimental infection of *Enseteventricosum* demonstrated that this important Ethiopian crop is highly susceptible to *Foc*-Race 2 (Ploetz, 2005). With the confirmation of *F.cugenangense* also present in representatives of infected ABB Tay banana cultivars, it is clear that Fusarium wilt caused by *Foc*-Race 2 is potentially more widespread than has often been assumed.

### ﻿*Fusariumcf.fujikuroi*; a new *Fusarium* lineage found within bananas

In addition to the *Fusarium* isolates collected from northern Vietnamese bananas belonging to FOSC, an infection with symptoms similar to *Foc* wilt was observed, yet the cultured isolate did not belong to FOSC. The morphological colony characteristics were comparable to those observed for FOSC cultures through having a pale purple colony reverse colour that became whitish towards the periphery with a dry colony surface appearance. However, when considering its phylogenetic position within the *Fusarium* genus, this isolate did not fall within *F.oxysporum* representatives, but was a member of the *F.fujikuroi* species complex (FFSC) where it is the sister lineage of *F.fujikuroi*. It is not uncommon that several *Fusarium* species cause the same disease pattern as this phenomenon has also been identified in mango deformity (Lima et al. 2009) and sugar beet wilt (Burlakoti et al. 2012). Within the FFSC, some species are known to be pathogenic for some *Musa* cultivars (*F.proliferatum*, *F.verticillioides*, *F.sacchari*, *F.lumajangense*, *F.desaboruense* and *F.musae*; Maldonado-Bonilla et al. 2019; Van Hove et al. 2002; Huang et al. 2019; Maryani et al. 2019b), yet to date, no other species of the FFSC - except for the above-mentioned - was identified as a pathogen for *Musa*. From a phylogenetic point of view, the novel *Fusarium* isolate that was obtained from the infected tissue of a triploid ABB Tay banana cultivar in Nam Dinh province is sister to *F.fujikuroi*. *Fusariumfujikuroi* is a widespread phytopathogen causing the bakanae disease in various *Oryzasativa* cultivars (rice), but it is also known to have a major impact on many other economically important crops (e.g., maize, wheat). This result increases our knowledge of the diversity of the *Fusarium* species that cause wilt symptoms on bananas. More importantly, it also demonstrates the urgent need to accurately identify plant pathogens that are morphologically very difficult to distinguish from each other in the field.

### ﻿*Fusarium* wilt on wild bananas

Although mainly observed on cultivated bananas, *Foc* has also been rarely recorded on wild *Musa* species (Ploetz and Pegg 2000). Waite (1963) noticed that Fusarium wilt also occurred on *M.acuminata*, *M.balbisiana*, *M.schizocarpa* and *M.textilis*. Since these specific *Musa* species belong to different sections in the genus - section Musa and Callimusa (Australimusa) - it is therefore likely that *Foc* can also infect other wild bananas. The current finding of a *Foc* isolate - phylogenetically situated within a *Foc*-Race 1 lineage - on a wild representative of *M.lutea*, could indicate that wild species are perhaps more susceptible to Fusarium wilt than previously assumed. Besides the visual symptoms of Fusarium wilt on this single specimen of *M.lutea*, none of the hundreds of individuals of *M.itinerans* and *M.lutea* surveyed in the same population showed any sign of *Foc* infection. This lack of visual symptoms either implies that F.oxysporumf.sp.cubense could have been absent from all those other wild accessions or that this *Fusarium* isolate related to *Foc*-Race 1 was present but failed to cause the disease in the other wild bananas. If the latter assumption is true, this could indicate that the pathogen has not necessarily co-evolved together with its host in Southeast Asia as postulated by Vakili (1965). Nevertheless, it is evident that *F.oxysporum* is omnipresent throughout the native distribution range of the *Musa* genus, and its infections take place when the plant is weakened due to external biotic or abiotic stressors, and the endophytic equilibrium is disturbed.

The current study demonstrates that bananas in northern Viet Nam which are infected by Fusarium wilt are characterised by a various range of *Fusarium* species (*F.cugenangense*, *F.odoratissimum*, *F.duoseptatum* and *F.tardichlamydosporum*) that belong to the *Fusariumoxysporum* species complex. Of these, the latter was most commonly present in cultivated bananas infected by Fusarium wilt, whereas the other species are less prominently present, yet in equal amounts. *Fusariumtardichlamydosporum* also occurred in a wild accession of *Musalutea*, indicating that wild bananas might function as a sink for *Foc*.

## Supplementary materials

Supplementary material 1 Table S1 Data type Docx file. Explanation note List of accessions used for the phylogenetic analyses, including voucher information and GenBank numbers. Asterisks indicate accessions for which new sequences were generated in the current study. https://binary.pensoft.net/file/644393This dataset is made available under the Open Database License (http://opendatacommons.org/licenses/odbl/1.0/). The Open Database License (ODbL) is a license agreement intended to allow users to freely share, modify, and use this Dataset while maintaining this same freedom for others, provided that the original source and author(s) are credited.Loan Le Thi, Arne Mertens, Dang Toan Vu, Dang Tuong Vu, Pham Le Anh Minh, Huy Nguyen Duc, Sander de Backer, Rony Swennen, Filip Vandelook, Bart Panis, Mario Amalfi, Cony Decock, Sofia I.F. Gomes, Vincent S.F.T. Merckx, Steven B. Janssens
