# Supplementary material for: ﻿Diversity of Fusarium associated banana wilt in northern Viet Nam
Source: MycoKeys. 2022 Feb 10;87:53–76. doi: 10.3897/mycokeys.87.72941 (PMC8854238; doi:10.3897/mycokeys.87.72941)
Supplement: Supplementary material 1 — Table S1 [file mycokeys-87-053-s001.docx]

**Supplementary Table S1.** List of accessions used for the phylogenetic analyses, including voucher information and GenBank numbers. Asterisks indicate accessions for which new sequences were generated in the current study.

| *Species* | Accession n° | *rpb1* | *rpb2* | *tef1a* |
| --- | --- | --- | --- | --- |
| *Albonectria albosuccinea* | NRRL 20459 | JX171585 | JX171585 | - |
| *Albonectria decemcellulare* | KNU01 | LC212975 | LC214751 | - |
| *Albonectria decemcellulare* | NRRL 13412 | JX171567 | JX171567 | - |
| *Bisifusarium delphinoides* | NRRL 36160 | HM347204 | HM347219 | - |
| *Bisifusarium dimerum* | NRRL 20691 | JX171478 | JX171592 | - |
| *Bisifusarium dimerum* | NRRL 36140 | HM347203 | HM347218 | - |
| *Bisifusarium domesticum* | NRRL 29976 | JX171528 | JX171641 | - |
| *Bisifusarium lunatum* | NRRL 36168 | JX171536 | JX171648 | - |
| *Bisifusarium nectrioides* | NRRL 20689 | JX171477 | JX171591 | - |
| *Bisifusarium penzigii* | NRRL 20711 | HM347202 | HM347217 | - |
| *Cosmospora arxii* | NRRL 54560 | JX171554 | JX171666 | - |
| *Cosmospora coccinea* | NRRL 53583 | JX171545 | JX171657 | - |
| *Cosmospora cymosum* | NRRL 54561 | JX171555 | JX171667 | - |
| *Cyanonectria cyanostoma* | NRRL 54603 | JX171553 | JX171665 | - |
| *Cyanonectria buxicola* | NRRL 36148 | JX171534 | HM068357 | - |
| *Cyanonectria cyanostoma* | NRRL 53998 | JX171546 | JX171658 | - |
| *Cylindrocarpon candidum* | NRRL 20485 | JX171588 | JX171474 | - |
| *Cylindrocarpon cylindroides* | NRRL 22505 | JX171499 | JX171612 | - |
| *Cylindrocarpon heteronema* | NRRL 20487 | JX171475 | JX171589 | - |
| *Cylindrocarpon sp.* | NRRL 6149 | JX171445 | JX171559 | - |
| *Fusarium acaciae/mearnsii* | NRRL 26755 | KM361640 | KM361658 | - |
| *Fusarium acuminatum* | NRRL 28449 | MG282373 | MG282402 | - |
| *Fusarium acuminatum* | NRRL 28652 | MG282384 | MG282414 | - |
| *Fusarium acutatum* | CBS 402.97 | MT010947 | KT154005 | MT010989 |
| *Fusarium acutatum* | NRRL 13308 | MN193911 | MN193883 | - |
| *Fusarium aethiopicum* | NRRL 46718 | KM361652 | KM361670 | - |
| *Fusarium agapanthi* | NRRL 31653 | KU900619 | KU900624 | - |
| *Fusarium agapanthi* | NRRL 54464 | KU900622 | KU900627 | - |
| *Fusarium algeriense* | NRRL 66647 | MF120488 | MT409451 | - |
| *Fusarium ambrosium* | NRRL 20438 | JX171470 | JX171584 | - |
| *Fusarium ambrosium* | NRRL 22345 | KC691586 | KC691618 | - |
| *Fusarium ambrosium* | NRRL 36510 | KC691588 | KC691619 | - |
| *Fusarium anguioides* | NRRL 25385 | JX171624 | JX171624 | - |
| *Fusarium anthophilum* | CBS 119858 | MT010940 | KU604275 | MT010997 |
| *Fusarium anthophilum* | NRRL 25214 | KU171696 | KF466403 | - |
| *Fusarium arcuatisporum* | NRRL 32997 | HM347164 | GQ505802 | - |
| *Fusarium armeniacum* | NRRL 43641 | HM347192 | GQ505494 | - |
| *Fusarium armeniacum* | NRRL 6227 | JX171446 | HQ154480 | - |
| *Fusarium arthrosporioides* | NRRL 26416 | MG282383 | MG282413 | - |
| *Fusarium asiaticum* | NRRL 13818 | JX171459 | JX171573 | - |
| *Fusarium astromatum* | NRRL 22566 | JX171500 | JX171613 | - |
| *Fusarium atrovinosum* | CBS 130394 | MN120714 | MN120734 | - |
| *Fusarium atrovinosum* | NRRL 13444 | JX171454 | JX171568 | - |
| *Fusarium atrovinosum* | NRRL 34016 | HM347170 | GQ505475 | - |
| *Fusarium austroafricanum* | NRRL 53441 | MH742536 | MH742615 | - |
| *Fusarium austroafricanum* | NRRL 66741 | MH742537 | MH742616 | - |
| *Fusarium austroamericanum* | NRRL 28585 | KM361643 | KM361661 | - |
| *Fusarium avenaceum* | INRA495 | MH667523 | MH667549 | - |
| *Fusarium avenaceum* | INRA496 | MH667524 | MH667550 | - |
| *Fusarium avenaceum* | NRRL 36374 | MG282366 | MG282395 | - |
| *Fusarium aywerte* | NRRL 25410 | JX171513 | JX171626 | - |
| *Fusarium babinda* | NRRL 25539 | JX171632 | JX171632 | - |
| *Fusarium babinda* | NRRL 53470 | MH742548 | MH742627 | - |
| *Fusarium babinda* | NRRL 53488 | MH742552 | MH742631 | - |
| *Fusarium bactridioides* | CBS 100057 | MT010939 | MT010963 | MT010995 |
| *Fusarium begoniae* | CBS 452.97 | MT010936 | MT010964 | MT010998 |
| *Fusarium begoniae* | NRRL 25300 | MN193914 | MN193886 | - |
| *Fusarium beomiforme* | NRRL 25174 | JX171506 | JX171619 | - |
| *Fusarium boothii* | NRRL 26916 | KM361641 | GQ915487 | - |
| *Fusarium brachygibbosum* | NRRL 13829 | JX171460 | JX171574 | - |
| *Fusarium brachygibbosum* | NRRL 31008 | JX171529 | MH845433 | - |
| *Fusarium brachygibbosum* | NRRL 34033 | HM347172 | GQ505482 | - |
| *Fusarium brasilicum* | NRRL 31238 | KM361645 | KM361663 | - |
| *Fusarium brevicatenulatum* | CBS 404.97 | MT010948 | MT010979 | MT011005 |
| *Fusarium brevicatenulatum* | NRRL 25447 | MN193915 | MN193887 | - |
| *Fusarium brevicaudatum* | NRRL 43694 | HM347193 | GQ505846 | - |
| *Fusarium buharicum* | NRRL 13371 | JX171449 | JX171563 | - |
| *Fusarium buharicum* | NRRL 25488 | KX302920 | KX302928 | - |
| *Fusarium bulbicola* | NRRL 13618 | KF466394 | KF466404 | - |
| *Fusarium bulbicola* | NRRL 22947 | KU171679 | KU171699 | - |
| *Fusarium burgessii* | NRRL 66654 | MF120495 | MT409450 | - |
| *Fusarium burgessii* | RBG5319 | KJ716217 | HQ646392 | - |
| *Fusarium caatingaense* | NRRL 34003 | HM347166 | GQ505805 | - |
| *Fusarium californicum* | BL24 | MK878580 | MK878565 | - |
| *Fusarium californicum* | BL28 | MK878582 | MK878567 | - |
| *Fusarium californicum* | BL30 | MK878584 | MK878569 | - |
| *Fusarium cerealis* | NRRL 13721 | KM361638 | KM361656 | - |
| *Fusarium cerealis* | NRRL 25491 | MG282371 | MG282400 | - |
| *Fusarium cf. fujikuroi* | Foc 58 | OM100607 | OM100611 | OM154157 |
| *Fusarium chlamydosporum* | CBS 145.25 | MN120715 | MN120735 | - |
| *Fusarium circinatum* | NRRL 25331 | JX171510 | HM068354 | - |
| *Fusarium citri* | MoPo1 | - | LT970750 | LT970778 |
| *Fusarium citri* | MoPo2 | - | LT970751 | LT970779 |
| *Fusarium citri* | MoSm29 | - | LT970754 | LT970782 |
| *Fusarium citricola* | CPC 27067 | LT746287 | LT746307 | LT746194 |
| *Fusarium citricola* | CPC 27069 | LT746288 | LT746308 | LT746195 |
| *Fusarium citricola* | CPC 27709 | LT746289 | LT746309 | LT746196 |
| *Fusarium clavum* | ITEM 10393 | - | LN901601 | LN901566 |
| *Fusarium clavum* | ITEM 10445 | - | LN901603 | LN901568 |
| *Fusarium clavum* | NRRL 34032 | HM347171 | GQ505813 | - |
| *Fusarium coffeatum* | CBS 635.76 | MN120717 | KU604328 | - |
| *Fusarium coicis* | RBG5368 | KP083269 | KP083274 | - |
| *Fusarium commune* | NRRL 28387 | JX171638 | HM068356 | - |
| *Fusarium compactum* | FiPo2=FiPoR | - | LT970748 | LT970776 |
| *Fusarium compactum* | NRRL 28029 | HM347150 | GQ505780 | - |
| *Fusarium concentricum* | CBS 450.97 | MT010942 | MT010981 | MT010992 |
| *Fusarium concolor* | NRRL 53455 | MH742506 | MH742583 | - |
| *Fusarium concolor* | NRRL 53480 | MH742513 | MH742591 | - |
| *Fusarium concolor* | NRRL 53493 | MH742535 | MH742614 | - |
| *Fusarium continuum* | F201030 | KM520387 | KM236782 | - |
| *Fusarium continuum* | F201127 | KM520386 | KM236779 | - |
| *Fusarium continuum* | F201129 | KM520385 | KM236781 | - |
| *Fusarium cortaderiae* | NRRL 29297 | KM361644 | KM361662 | - |
| *Fusarium cugenangense* | InaCC F983 | LS479559 | LS479307 | LS479756 |
| *Fusarium cugenangense* | InaCC F984 | LS479560 | LS479308 | LS479757 |
| *Fusarium cugenangense* | NRRL 25433 | LS479462 | LS479202 | LS479648 |
| *Fusarium cugenangense* | NRRL 36118 (VCG01221) | LS479477 | LS479221 | LS479669 |
| *Fusarium cugenangense* | BRIP29094 | KX434922 | KX434957 | - |
| *Fusarium cugenangense* | BRIP45952 | KX434923 | KX434958 | - |
| *Fusarium cugenangense* | NRRL 25387 | JX171625 | HM347209 | - |
| *Fusarium cugenangense* | Foc 10 | - | OM100625 | OM154153 |
| *Fusarium cugenangense* | Foc 4 | - | OM100626 | OM154154 |
| *Fusarium culmorum* | NRRL 25475 | JX171515 | JX171628 | - |
| *Fusarium culmorum* | NRRL 66294 | MG282380 | MG282410 | - |
| *Fusarium dactylidis* | NRRL 29298 | KM361654 | KM361672 | - |
| *Fusarium dactylidis* | NRRL 29380 | KM361653 | KM361671 | - |
| *Fusarium denticulatum* | CBS 407.97 | MT010953 | MT010970 | MT011002 |
| *Fusarium dlaminii* | NRRL 13164 | KU171681 | KU171701 | - |
| *Fusarium duoseptatum* | FocMal43 (VCG01217) | - | LS479207 | LS479653 |
| *Fusarium duoseptatum* | InaCC F828 | LS479520 | LS479266 | LS479715 |
| *Fusarium duoseptatum* | InaCC F829 | LS479528 | LS479274 | LS479723 |
| *Fusarium duoseptatum* | InaCC F831 | LS479538 | LS479285 | LS479734 |
| *Fusarium duoseptatum* | InaCC F835 | LS479567 | LS479315 | LS479764 |
| *Fusarium duoseptatum* | InaCC F911 | - | LS479234 | LS479683 |
| *Fusarium duoseptatum* | InaCC F915 | LS479494 | LS479238 | LS479687 |
| *Fusarium duoseptatum* | InaCC F916 | LS479495 | LS479239 | LS479688 |
| *Fusarium duoseptatum* | InaCC F920 | LS479499 | LS479244 | LS479693 |
| *Fusarium duoseptatum* | InaCC F921 | LS479500 | LS479245 | LS479694 |
| *Fusarium duoseptatum* | InaCC F975 | LS479549 | LS479296 | LS479745 |
| *Fusarium duoseptatum* | InaCC F976 | LS479550 | LS479297 | LS479746 |
| *Fusarium duoseptatum* | InaCC F977 | LS479551 | LS479298 | LS479747 |
| *Fusarium duoseptatum* | InaCC F978 | LS479552 | LS479299 | LS479748 |
| *Fusarium duoseptatum* | InaCC F979 | LS479553 | LS479300 | LS479749 |
| *Fusarium duoseptatum* | Indo80 | LS479619 | LS479387 | LS479829 |
| *Fusarium duoseptatum* | NRRL 36115 (VCG01224) | LS479475 | LS479218 | LS479666 |
| *Fusarium duoseptatum* | NRRL 36116 (VCG01223) | - | LS479219 | LS479667 |
| *Fusarium duoseptatum* | Foc 38 | - | OM100624 | OM154152 |
| *Fusarium equiseti* | FUS18 | MN692709 | MN692731 | - |
| *Fusarium equiseti* | FUS28 | MN692714 | MN692736 | - |
| *Fusarium equiseti* | FUS42 | MN692718 | MN692740 | - |
| *Fusarium equiseti* | ITEM 10675 | - | LN901607 | LN901573 |
| *Fusarium equiseti* | ITEM 11363 | - | LN901609 | LN901574 |
| *Fusarium equiseti* | NRRL 43636 | HM347189 | GQ505841 | - |
| *Fusarium ficicrescens* | CBS 125178 | MT010950 | KT154002 | MT011004 |
| *Fusarium flagelliforme* | ITEM 11296 | - | LN901606 | LN901572 |
| *Fusarium flocciferum* | NRRL 25473 | JX171514 | JX171627 | - |
| *Fusarium flocciferum* | NRRL 45999 | HM347195 | GQ505497 | - |
| *Fusarium foetens* | CBS 110286 | MT010945 | MT010984 | MT011001 |
| *Fusarium foetens* | NRRL 38302 | JX171652 | JX171652 | - |
| *Fusarium fujikuroi* | NRRL 13566 | JX171456 | JX171570 | - |
| *Fusarium fujikuroi* | NRRL 5538 | MN193916 | MN193888 | - |
| *Fusarium fujikuroi* | NRRL 66288 | MG282385 | MG282415 | - |
| *Fusarium gaditjirrii* | NRRL 45417 | JX171654 | JX171654 | - |
| *Fusarium globosum* | NRRL 26132 | LT746301 | LT746343 | - |
| *Fusarium globosum* | NRRL 26133 | LT746302 | LT746344 | - |
| *Fusarium globosum* | NRRL 26134 | LT746303 | LT746345 | - |
| *Fusarium gracilipes* | NRRL 43635 | HM347188 | GQ505840 | - |
| *Fusarium graminearum* | SP100 | - | MN625698 | MK611901 |
| *Fusarium graminearum* | SP102 | - | MN625699 | MK611900 |
| *Fusarium graminearum* | SP99 | - | MN625697 | MK611899 |
| *Fusarium graminum* | NRRL 20692 | JX171479 | JX171593 | - |
| *Fusarium grosmichelii* | InaCC F832 | LS479542 | LS479289 | LS479738 |
| *Fusarium grosmichelii* | InaCC F833 | LS479548 | LS479295 | LS479744 |
| *Fusarium grosmichelii* | InaCC F848 | LS479588 | LS479338 | LS479786 |
| *Fusarium grosmichelii* | InaCC F849 | LS479589 | LS479339 | LS479787 |
| *Fusarium grosmichelii* | InaCC F850 | - | LS479340 | LS479788 |
| *Fusarium grosmichelii* | InaCC F851 | - | LS479341 | LS479789 |
| *Fusarium grosmichelii* | InaCC F852 | - | LS479342 | LS479790 |
| *Fusarium grosmichelii* | InaCC F853 | - | LS479343 | LS479791 |
| *Fusarium grosmichelii* | InaCC F854 | LS479591 | LS479345 | LS479793 |
| *Fusarium grosmichelii* | InaCC F855 | LS479592 | LS479346 | LS479794 |
| *Fusarium grosmichelii* | InaCC F861 | LS479597 | LS479351 | LS479797 |
| *Fusarium grosmichelii* | InaCC F862 | LS479598 | LS479352 | LS479798 |
| *Fusarium grosmichelii* | InaCC F863 | LS479599 | LS479353 | LS479799 |
| *Fusarium grosmichelii* | InaCC F867 | - | LS479360 | LS479806 |
| *Fusarium grosmichelii* | InaCC F868 | - | LS479361 | LS479807 |
| *Fusarium grosmichelii* | InaCC F884 | LS479616 | LS479382 | LS479824 |
| *Fusarium grosmichelii* | InaCC F887 | LS479620 | LS479388 | LS479830 |
| *Fusarium grosmichelii* | InaCC F888 | LS479621 | LS479389 | LS479831 |
| *Fusarium grosmichelii* | Indo83 | - | LS479390 | - |
| *Fusarium grosmichelii* | NRRL 36120 (VCG01218) | LS479478 | LS479222 | LS479670 |
| *Fusarium guilinense* | NRRL 32865 | HM347161 | GQ505792 | - |
| *Fusarium guttiforme* | CBS 409.97 | MT010938 | MT010967 | MT010999 |
| *Fusarium guttiforme* | NRRL 2294 | MN193917 | MN193889 | - |
| *Fusarium guttiforme* | NRRL 22945 | JX171505 | JX171618 | - |
| *Fusarium hainanense* | NRRL 26417 | JX171522 | GQ505776 | - |
| *Fusarium heterosporum* | NRRL 20693 | JX171480 | JX171594 | - |
| *Fusarium hexaseptatum* | InaCC F866 | - | LS479359 | LS479805 |
| *Fusarium hostae* | NRRL 29888 | MT409435 | MT409445 | - |
| *Fusarium hostae* | NRRL 29889 | JX171640 | JX171640 | - |
| *Fusarium humicola* | CBS 124.73 | MN120718 | MN120738 | - |
| *Fusarium incarnatum* | ITEM 6748 | - | LN901618 | LN901582 |
| *Fusarium incarnatum* | ITEM 7155 | - | LN901617 | LN901581 |
| *Fusarium incarnatum* | NRRL 32866 | HM347162 | GQ505793 | - |
| *Fusarium ipomoeae* | NRRL 43640 | HM347191 | GQ505845 | - |
| *Fusarium irregulare* | NRRL 32175 | JX171532 | GQ505787 | - |
| *Fusarium irregulare* | NRRL 34006 | HM347169 | GQ505808 | - |
| *Fusarium kalimantanense* | InaCC F917 | LS479497 | LS479241 | LS479690 |
| *Fusarium kalimantanense* | InaCC F918 | - | LS479242 | LS479691 |
| *Fusarium kalimantanense* | InaCC F922 | - | LS479246 | LS479695 |
| *Fusarium kyushuense* | NRRL 66296 | MG282364 | MG282393 | - |
| *Fusarium lacertarum* | NRRL 20423 | JX171581 | GQ505771 | - |
| *Fusarium lactis* | CBS 411.97 | MT010954 | MT010969 | MT011010 |
| *Fusarium langsethiae* | NRRL 54940 | JX171550 | JX171662 | - |
| *Fusarium lateritium* | NRRL 13622 | JX171457 | HM068350 | - |
| *Fusarium lateritium* | NRRL 25197 | HM347140 | HM347207 | - |
| *Fusarium longipes* | NRRL 13368 | JX171448 | JX171562 | - |
| *Fusarium longipes* | NRRL 13374 | JX171450 | JX171564 | - |
| *Fusarium longipes* | NRRL 20723 | JX171483 | JX171596 | - |
| *Fusarium luffae* | NRRL 32522 | HM347158 | GQ505790 | - |
| *Fusarium lunulosporum* | NRRL 13393 | KM361637 | KM361655 | - |
| *Fusarium lyarnte* | NRRL 54252 | JX171661 | MN193908 | - |
| *Fusarium mangiferae* | MUCL 54671 | - | LT575059 | LT574978 |
| *Fusarium mangiferae* | NRRL 25226 | JX171509 | HM068353 | - |
| *Fusarium mangiferae* | UMAF 910 | KP753434 | KP753441 | - |
| *Fusarium meridionale* | NRRL 28436 | KM361642 | KM361660 | - |
| *Fusarium mesoamericanum* | NRRL 25797 | KM361639 | KM361657 | - |
| *Fusarium mexicanum* | MICMW 32.13a | MN242900 | MN724975 | - |
| *Fusarium mexicanum* | MICMW 3A | MN242905 | MN724980 | - |
| *Fusarium miscanthi* | NRRL 26231 | JX171634 | JX171634 | - |
| *Fusarium multiceps* | NRRL 43639 | HM347190 | GQ505844 | - |
| *Fusarium mundagurra* | RBG5717 | KP083272 | KP083276 | - |
| *Fusarium musae* | CBS 624.87 | MT010957 | MT010973 | MT010991 |
| *Fusarium nanum* | NRRL 32868 | HM347163 | GQ505795 | - |
| *Fusarium napiforme* | CBS 748.97 | MT010958 | KU604233 | MT011011 |
| *Fusarium napiforme* | F111 | KU974338 | KU974364 | - |
| *Fusarium napiforme* | NRRL 25196 | MN193919 | MN193891 | - |
| *Fusarium nelsonii* | NRRL 13338 | JX171447 | GQ505466 | - |
| *Fusarium newnesense* | RBG5443 | KJ397218 | KJ397254 | - |
| *Fusarium nisikadoi* | NRRL 25179 | JX171620 | JX171620 | - |
| *Fusarium nisikadoi* | NRRL 25203 | MG282388 | MG282418 | - |
| *Fusarium nisikadoi* | NRRL 25308 | MG282391 | MG282421 | - |
| *Fusarium nodosum* | CBS 200.63 | MN120724 | MN120742 | - |
| *Fusarium nodosum* | CBS 201.63 | MN120725 | MN120743 | - |
| *Fusarium nodosum* | CBS 698.74 | MN120726 | MN120744 | - |
| *Fusarium nurragi* | NRRL 36452 | JX171538 | JX171650 | - |
| *Fusarium nygamai* | CBS 749.97 | MT010955 | KU604262 | MT011009 |
| *Fusarium nygamai* | NRRL 66291 | MG282368 | MG282397 | - |
| *Fusarium nygamai* | NRRL 66293 | MG282367 | MG282396 | - |
| *Fusarium odoratissimum* | InaCC F1000 | LS479575 | LS479323 | LS479772 |
| *Fusarium odoratissimum* | InaCC F816 | LS479485 | LS479228 | LS479677 |
| *Fusarium odoratissimum* | InaCC F817 | LS479556 | LS479304 | LS479753 |
| *Fusarium odoratissimum* | InaCC F818 | LS479584 | LS479333 | LS479782 |
| *Fusarium odoratissimum* | InaCC F819 | LS479600 | LS479354 | LS479800 |
| *Fusarium odoratissimum* | InaCC F821 | LS479609 | LS479374 | LS479818 |
| *Fusarium odoratissimum* | InaCC F822 | LS479618 | LS479386 | LS479828 |
| *Fusarium odoratissimum* | InaCC F824 | LS479486 | LS479229 | LS479678 |
| *Fusarium odoratissimum* | InaCC F825 | LS479496 | LS479240 | LS479689 |
| *Fusarium odoratissimum* | InaCC F836 | LS479577 | LS479325 | LS479774 |
| *Fusarium odoratissimum* | InaCC F837 | LS479578 | LS479326 | LS479775 |
| *Fusarium odoratissimum* | InaCC F838 | LS479579 | LS479327 | LS479776 |
| *Fusarium odoratissimum* | InaCC F839 Indo25 (VCG01219) | LS479580 | LS479328 | LS479777 |
| *Fusarium odoratissimum* | InaCC F840 | - | LS479329 | LS479778 |
| *Fusarium odoratissimum* | InaCC F846 | - | LS479336 | LS479785 |
| *Fusarium odoratissimum* | InaCC F857 | LS479594 | LS479348 | LS479795 |
| *Fusarium odoratissimum* | InaCC F864 | - | LS479356 | LS479802 |
| *Fusarium odoratissimum* | InaCC F870 | LS479602 | LS479363 | LS479809 |
| *Fusarium odoratissimum* | InaCC F871 | - | LS479365 | LS479811 |
| *Fusarium odoratissimum* | InaCC F873 | LS479604 | LS479369 | LS479814 |
| *Fusarium odoratissimum* | InaCC F875 | LS479607 | LS479372 | LS479816 |
| *Fusarium odoratissimum* | InaCC F876 | LS479608 | LS479373 | LS479817 |
| *Fusarium odoratissimum* | InaCC F877 | LS479610 | LS479375 | LS479819 |
| *Fusarium odoratissimum* | InaCC F879 | LS479612 | LS479377 | LS479820 |
| *Fusarium odoratissimum* | InaCC F880 | - | LS479378 | LS479821 |
| *Fusarium odoratissimum* | InaCC F882 | LS479614 | LS479380 | LS479822 |
| *Fusarium odoratissimum* | InaCC F883 | LS479615 | LS479381 | LS479823 |
| *Fusarium odoratissimum* | InaCC F885 | - | LS479384 | LS479826 |
| *Fusarium odoratissimum* | InaCC F891 | - | LS479393 | LS479833 |
| *Fusarium odoratissimum* | InaCC F892 | LS479624 | LS479394 | LS479834 |
| *Fusarium odoratissimum* | InaCC F893 | LS479625 | LS479395 | LS479835 |
| *Fusarium odoratissimum* | InaCC F894 | LS479626 | LS479396 | LS479836 |
| *Fusarium odoratissimum* | InaCC F896 | LS479629 | LS479399 | LS479839 |
| *Fusarium odoratissimum* | InaCC F897 | LS479630 | LS479400 | LS479840 |
| *Fusarium odoratissimum* | InaCC F898 | LS479631 | LS479401 | LS479841 |
| *Fusarium odoratissimum* | InaCC F899 | LS479632 | LS479402 | LS479842 |
| *Fusarium odoratissimum* | InaCC F900 | LS479633 | LS479403 | LS479843 |
| *Fusarium odoratissimum* | InaCC F901 | LS479634 | LS479404 | LS479844 |
| *Fusarium odoratissimum* | InaCC F902 | LS479635 | LS479405 | LS479845 |
| *Fusarium odoratissimum* | InaCC F903 | LS479636 | LS479406 | LS479846 |
| *Fusarium odoratissimum* | InaCC F904 | LS479637 | LS479407 | LS479847 |
| *Fusarium odoratissimum* | InaCC F905 | LS479638 | LS479408 | LS479848 |
| *Fusarium odoratissimum* | InaCC F906 | LS479639 | LS479409 | LS479849 |
| *Fusarium odoratissimum* | InaCC F907 | LS479487 | LS479230 | LS479679 |
| *Fusarium odoratissimum* | InaCC F908 | LS479488 | LS479231 | LS479680 |
| *Fusarium odoratissimum* | InaCC F909 | LS479489 | LS479232 | LS479681 |
| *Fusarium odoratissimum* | InaCC F910 | LS479490 | LS479233 | LS479682 |
| *Fusarium odoratissimum* | InaCC F912 | LS479491 | LS479235 | LS479684 |
| *Fusarium odoratissimum* | InaCC F919 | LS479498 | LS479243 | LS479692 |
| *Fusarium odoratissimum* | InaCC F923 | LS479501 | LS479247 | LS479696 |
| *Fusarium odoratissimum* | InaCC F924 | LS479502 | LS479248 | LS479697 |
| *Fusarium odoratissimum* | InaCC F925 | LS479503 | LS479249 | LS479698 |
| *Fusarium odoratissimum* | InaCC F926 | LS479504 | LS479250 | LS479699 |
| *Fusarium odoratissimum* | InaCC F927 | LS479506 | LS479252 | LS479701 |
| *Fusarium odoratissimum* | InaCC F928 | LS479507 | LS479253 | LS479702 |
| *Fusarium odoratissimum* | InaCC F929 | LS479508 | LS479254 | LS479703 |
| *Fusarium odoratissimum* | InaCC F930 | LS479509 | LS479255 | LS479704 |
| *Fusarium odoratissimum* | InaCC F931 | LS479510 | LS479256 | LS479705 |
| *Fusarium odoratissimum* | InaCC F932 | LS479511 | LS479257 | LS479706 |
| *Fusarium odoratissimum* | InaCC F933 | LS479512 | LS479258 | LS479707 |
| *Fusarium odoratissimum* | InaCC F934 | LS479514 | LS479260 | LS479709 |
| *Fusarium odoratissimum* | InaCC F935 | LS479515 | LS479261 | LS479710 |
| *Fusarium odoratissimum* | InaCC F936 | LS479516 | LS479262 | LS479711 |
| *Fusarium odoratissimum* | InaCC F937 | LS479517 | LS479263 | LS479712 |
| *Fusarium odoratissimum* | InaCC F938 | LS479518 | LS479264 | LS479713 |
| *Fusarium odoratissimum* | InaCC F939 | LS479519 | LS479265 | LS479714 |
| *Fusarium odoratissimum* | InaCC F942 | LS479521 | LS479267 | LS479716 |
| *Fusarium odoratissimum* | InaCC F943 | LS479522 | LS479268 | LS479717 |
| *Fusarium odoratissimum* | InaCC F944 | LS479523 | LS479269 | LS479718 |
| *Fusarium odoratissimum* | InaCC F945 | LS479524 | LS479270 | LS479719 |
| *Fusarium odoratissimum* | InaCC F946 | LS479525 | LS479271 | LS479720 |
| *Fusarium odoratissimum* | InaCC F947 | LS479526 | LS479272 | LS479721 |
| *Fusarium odoratissimum* | InaCC F948 | LS479527 | LS479273 | LS479722 |
| *Fusarium odoratissimum* | InaCC F953 | LS479529 | LS479275 | LS479724 |
| *Fusarium odoratissimum* | InaCC F954 | LS479530 | LS479276 | LS479725 |
| *Fusarium odoratissimum* | InaCC F955 | LS479531 | LS479277 | LS479726 |
| *Fusarium odoratissimum* | InaCC F973 | LS479547 | LS479294 | LS479743 |
| *Fusarium odoratissimum* | InaCC F985 | LS479562 | LS479310 | LS479759 |
| *Fusarium odoratissimum* | InaCC F986 | LS479563 | LS479311 | LS479760 |
| *Fusarium odoratissimum* | InaCC F989 | LS479566 | LS479314 | LS479763 |
| *Fusarium odoratissimum* | InaCC F990 | LS479568 | LS479316 | LS479765 |
| *Fusarium odoratissimum* | InaCC F994 | LS479569 | LS479317 | LS479766 |
| *Fusarium odoratissimum* | InaCC F997 | LS479572 | LS479320 | LS479769 |
| *Fusarium odoratissimum* | InaCC F998 | LS479573 | LS479321 | LS479770 |
| *Fusarium odoratissimum* | InaCC F999 | LS479574 | LS479322 | LS479771 |
| *Fusarium odoratissimum* | Indo204 | LS479561 | LS479309 | LS479758 |
| *Fusarium odoratissimum* | Indo222 | LS479576 | LS479324 | LS479773 |
| *Fusarium odoratissimum* | Indo4 | LS479590 | LS479344 | LS479792 |
| *Fusarium odoratissimum* | Indo53 | - | LS479357 | LS479803 |
| *Fusarium odoratissimum* | Indo61 | - | LS479366 | LS479812 |
| *Fusarium odoratissimum* | Indo62 | - | LS479367 | - |
| *Fusarium odoratissimum* | Indo66 | LS479605 | LS479370 | LS479815 |
| *Fusarium odoratissimum* | Indo77 | LS479617 | LS479383 | LS479825 |
| *Fusarium odoratissimum* | Indo89 | LS479627 | LS479397 | LS479837 |
| *Fusarium odoratissimum* | JV11 | LS479465 | LS479205 | LS479651 |
| *Fusarium odoratissimum* | Leb1.2C | LS479466 | LS479206 | LS479652 |
| *Fusarium odoratissimum* | NRRL 36102 (VCG0121) | LS479468 | LS479209 | LS479655 |
| *Fusarium odoratissimum* | Pak1.1A | LS479479 | LS479223 | LS479671 |
| *Fusarium odoratissimum* | Foc 56 | OM100606 | OM100610 | OM154155 |
| *Fusarium odoratissimum* | Foc 61 | - | OM100627 | OM154156 |
| *Fusarium odoratissimum* | FocII5 (VCG01213) | LS479459 | LS479198 | LS479644 |
| *Fusarium pernambucanum* | NRRL 32864 | HM347160 | GQ505791 | - |
| *Fusarium peruvianum* | CBS 511.75 | MN120728 | MN120746 | - |
| *Fusarium petersiae* | JW14004 | MG386138 | MG386149 | - |
| *Fusarium petersiae* | JW14005 | MG386139 | MG386150 | - |
| *Fusarium phialophorum* | FocIndo25 | LS479464 | LS479204 | LS479650 |
| *Fusarium phialophorum* | FocST4.98 (VCG0120) | LS479484 | LS479227 | LS479676 |
| *Fusarium phialophorum* | InaCC F826 | LS479505 | LS479251 | LS479700 |
| *Fusarium phialophorum* | InaCC F827 | LS479513 | LS479259 | LS479708 |
| *Fusarium phialophorum* | InaCC F830 | LS479536 | LS479282 | LS479731 |
| *Fusarium phialophorum* | InaCC F834 | LS479557 | LS479305 | LS479754 |
| *Fusarium phialophorum* | InaCC F842 | LS479582 | LS479331 | LS479780 |
| *Fusarium phialophorum* | InaCC F843 | LS479583 | LS479332 | LS479781 |
| *Fusarium phialophorum* | InaCC F844 | LS479585 | LS479334 | LS479783 |
| *Fusarium phialophorum* | InaCC F845 | LS479586 | LS479335 | LS479784 |
| *Fusarium phialophorum* | InaCC F889 Indo84 (VCG01216) | LS479622 | LS479391 | LS479832 |
| *Fusarium phialophorum* | InaCC F969 | LS479543 | LS479290 | LS479739 |
| *Fusarium phialophorum* | InaCC F970 | LS479544 | LS479291 | LS479740 |
| *Fusarium phialophorum* | InaCC F971 | LS479545 | LS479292 | LS479741 |
| *Fusarium phialophorum* | InaCC F972 | LS479546 | LS479293 | LS479742 |
| *Fusarium phialophorum* | InaCC F981 | - | LS479303 | LS479752 |
| *Fusarium phialophorum* | InaCC F982 | LS479558 | LS479306 | LS479755 |
| *Fusarium phialophorum* | InaCC F987 | LS479564 | LS479312 | LS479761 |
| *Fusarium phialophorum* | InaCC F995 | LS479570 | LS479318 | LS479767 |
| *Fusarium phialophorum* | InaCC F996 | LS479571 | LS479319 | LS479768 |
| *Fusarium phialophorum* | NRRL 36101 (VCG0123) | LS479467 | LS479208 | LS479654 |
| *Fusarium phialophorum* | NRRL 36103 (VCG0122) | LS479469 | LS479210 | LS479656 |
| *Fusarium phialophorum* | NRRL 36109 (VCG01211) | LS479471 | LS479214 | LS479661 |
| *Fusarium phialophorum* | NRRL 36112 (VCG01215) | LS479473 | LS479216 | LS479664 |
| *Fusarium phialophorum* | R1.0124 | LS479483 | - | LS479675 |
| *Fusarium phyllophilum* | NRRL 13617 | KF466399 | KF466410 | - |
| *Fusarium poae* | NRRL 13714 | JX171458 | JX171572 | - |
| *Fusarium poae* | NRRL 66297 | MG282363 | MG282392 | - |
| *Fusarium praegraminearum* | NRRL 39664 | KX260125 | KX260126 | - |
| *Fusarium proliferatum* | ITEM2287 | LT841251 | LT841252 | LT841245 |
| *Fusarium proliferatum* | ITEM2400 | LT841265 | LT841266 | LT841259 |
| *Fusarium proliferatum* | NRRL 22944 | JX171504 | HM068352 | - |
| *Fusarium pseudoanthophilum* | CBS 414.97 | MT010949 | MT010980 | MT011006 |
| *Fusarium pseudocircinatum* | NRRL 22946 | MG838070 | MN724939 | - |
| *Fusarium pseudocircinatum* | NRRL 31631 | MG838073 | MN724942 | - |
| *Fusarium pseudocircinatum* | NRRL 53570 | MG838075 | MN724944 | - |
| *Fusarium pseudograminearum* | NRRL 28062 | JX171524 | JX171637 | - |
| *Fusarium pseudograminearum* | NRRL 28065 | MG282389 | MG282419 | - |
| *Fusarium pseudonygamai* | CBS 417.97 | MT010951 | MT010978 | MT011008 |
| *Fusarium purpurascens* | InaCC F823 | LS479628 | LS479398 | LS479838 |
| *Fusarium purpurascens* | InaCC F886 | - | LS479385 | LS479827 |
| *Fusarium purpurascens* | InaCC F913 | LS479492 | LS479236 | LS479685 |
| *Fusarium purpurascens* | InaCC F914 | LS479493 | LS479237 | LS479686 |
| *Fusarium purpurascens* | InaCC F966 | LS479539 | LS479286 | LS479735 |
| *Fusarium purpurascens* | InaCC F967 | LS479540 | LS479287 | LS479736 |
| *Fusarium purpurascens* | InaCC F968 | LS479541 | LS479288 | LS479737 |
| *Fusarium purpurascens* | NRRL 36107 (VCG0126) | - | LS479213 | LS479659 |
| *Fusarium ramigenum* | CBS 418.97 | MT010959 | MT010975 | MT011012 |
| *Fusarium ramigenum* | NRRL 25208 | KF466401 | KF466412 | - |
| *Fusarium redolens* | CBS 743.97 | MT010935 | MT010961 | MT010987 |
| *Fusarium redolens* | NRRL 22901 | JX171616 | JX171616 | - |
| *Fusarium redolens* | NRRL 25600 | MT409433 | MT409443 | - |
| *Fusarium sacchari* | CBS 147.25 | MT010941 | MT010962 | MT010988 |
| *Fusarium sacchari* | NRRL 44901 | HM347194 | HM347212 | - |
| *Fusarium sacchari* | YN BS37 | MK983434 | MK829737 | - |
| *Fusarium salinense* | CPC 26403 | LT746284 | LT746304 | LT746191 |
| *Fusarium salinense* | CPC 26457 | LT746285 | LT746305 | LT746192 |
| *Fusarium salinense* | CPC 26973 | LT746286 | LT746306 | LT746193 |
| *Fusarium sambucinum* | NRRL 22187 | JX171493 | JX171606 | - |
| *Fusarium sangayamense* | InaCC F961 | - | LS479284 | LS479733 |
| *Fusarium sarcochroum* | CPC 28075 | LT746296 | LT746324 | LT746211 |
| *Fusarium sarcochroum* | CPC 28116 | LT746297 | LT746325 | LT746212 |
| *Fusarium sarcochroum* | NRRL 20472 | JX171472 | JX171586 | - |
| *Fusarium scirpi* | NRRL 13402 | JX171452 | GQ505770 | - |
| *Fusarium siculi* | CPC 27188 | LT746299 | LT746327 | LT746214 |
| *Fusarium siculi* | CPC 27189 | LT746300 | LT746328 | LT746215 |
| *Fusarium spinosum* | CBS 122438 | MN120729 | MN120747 | - |
| *Fusarium spinosum* | NRRL 43631 | HM347187 | GQ505491 | - |
| *Fusarium sporodochiale* | CBS 199.63 | MN120730 | MN120748 | - |
| *Fusarium sporodochiale* | CBS 220.61 | MN120731 | MN120749 | - |
| *Fusarium sporotrichioides* | NRRL 25479 | HM347144 | HM347210 | - |
| *Fusarium sporotrichioides* | NRRL 3299 | JX171444 | DQ676587 | - |
| *Fusarium sporotrichioides* | NRRL 66295 | MG282378 | MG282408 | - |
| *Fusarium sterilihyphosum* | NRRL 25623 | LR792581 | LR792617 | - |
| *Fusarium stilboides* | HA 1 2 | MK887361 | MK887362 | - |
| *Fusarium stilboides* | NRRL 20429 | JX171468 | JX171582 | - |
| *Fusarium subglutinans* | NRRL 22016 | JX171486 | JX171599 | - |
| *Fusarium subglutinans* | NRRL 54158 | HM347201 | HM347216 | - |
| *Fusarium subglutinans* | NRRL 66333 | MN193926 | MN193898 | - |
| *Fusarium sublunatum* | NRRL 13384 | JX171451 | JX171565 | - |
| *Fusarium subtropicale* | NRRL 66764 | MH706972 | MH706973 | - |
| *Fusarium sulawesiense* | NRRL 34004 | HM347167 | GQ505806 | - |
| *Fusarium tanahbumbuense* | CBS 101138 | MN120733 | MN120751 | - |
| *Fusarium tanahbumbuense* | NRRL 34005 | HM347168 | GQ505807 | - |
| *Fusarium tardichlamydosporum* | BRIP44611 | KX434927 | KX434962 | - |
| *Fusarium tardichlamydosporum* | BRIP62955 | KX434936 | KX434971 | - |
| *Fusarium tardichlamydosporum* | Foc 1 | - | OM100620 | OM154148 |
| *Fusarium tardichlamydosporum* | Foc 11 | - | OM100614 | OM154142 |
| *Fusarium tardichlamydosporum* | Foc 16 | - | OM100621 | OM154149 |
| *Fusarium tardichlamydosporum* | Foc 18 | - | OM100613 | OM154141 |
| *Fusarium tardichlamydosporum* | Foc 2 | - | OM100623 | OM154151 |
| *Fusarium tardichlamydosporum* | Foc 21 | - | OM100618 | OM154146 |
| *Fusarium tardichlamydosporum* | Foc 23-2 | - | OM100622 | OM154150 |
| *Fusarium tardichlamydosporum* | Foc 24 | OM100605 | OM100609 | OM154139 |
| *Fusarium tardichlamydosporum* | Foc 25-1 | - | OM100612 | OM154140 |
| *Fusarium tardichlamydosporum* | Foc 25-2 | - | OM100615 | OM154143 |
| *Fusarium tardichlamydosporum* | Foc 5 | - | OM100619 | OM154147 |
| *Fusarium tardichlamydosporum* | Foc 6 | - | OM100616 | OM154144 |
| *Fusarium tardichlamydosporum* | Foc 7 | - | OM100617 | OM154145 |
| *Fusarium tardichlamydosporum* | InaCC F956 | LS479532 | LS479278 | LS479727 |
| *Fusarium tardichlamydosporum* | InaCC F957 | LS479533 | LS479279 | LS479728 |
| *Fusarium tardichlamydosporum* | InaCC F958 | LS479534 | LS479280 | LS479729 |
| *Fusarium tardichlamydosporum* | InaCC F959 | LS479535 | LS479281 | LS479730 |
| *Fusarium tardichlamydosporum* | NRRL 36105 (VCG0124) | LS479470 | LS479211 | LS479657 |
| *Fusarium tardichlamydosporum* | NRRL 36106 (VCG0125) | - | LS479212 | LS479658 |
| *Fusarium tardichlamydosporum* | NRRL 36111 (VCG0128) | LS479472 | LS479215 | LS479663 |
| *Fusarium tardichlamydosporum* | NRRL 36117 (VCG01222) | LS479476 | LS479220 | LS479668 |
| *Fusarium tardicrescens* | NRRL 36113 (VCG01214) | LS479474 | LS479217 | LS479665 |
| *Fusarium tardicrescens* | NRRL 37622 | LS479463 | LS479203 | LS479649 |
| *Fusarium tardicrescens* | NRRL 54005 | LS479482 | LS479226 | LS479674 |
| *Fusarium tardicrescens* | NRRL 54008 | LS479481 | LS479225 | LS479673 |
| *Fusarium temperatum* | NRRL 25622 | LR792582 | LR792618 | - |
| *Fusarium thapsinum* | NRRL 22045 | JX171487 | JX171600 | - |
| *Fusarium thapsinum* | NRRL 22049 | KU171713 | MN193899 | - |
| *Fusarium tjaetaba* | RBG5361 | KP083267 | KP083275 | - |
| *Fusarium torreyae* | NRRL 54149 | JX171548 | HM068359 | - |
| *Fusarium torulosum* | NRRL 22748 | JX171502 | JX171615 | - |
| *Fusarium torulosum* | NRRL 52772 | JF741003 | MH582377 | - |
| *Fusarium tricinctum* | NRRL 25481 | JX171516 | HM068327 | - |
| *Fusarium tupiense* | NRRL 53984 | LR792583 | LR792619 | - |
| *Fusarium tupiense* | UMAF 0917 | KP753436 | KP753443 | - |
| *Fusarium tupiense* | UMAF 0933 | KP753437 | KP753444 | - |
| *Fusarium udum* | NRRL 25194 | MN193928 | MN193900 | - |
| *Fusarium ussurianum* | NRRL 45681 | KM361648 | KM361666 | - |
| *Fusarium venenatum* | NRRL 22196 | JX171494 | JX171607 | - |
| *Fusarium verticillioides* | YN DH24 | MK886821 | MK886821 | - |
| *Fusarium verticillioides* | YN DH28 | MK983460 | MK983384 | - |
| *Fusarium verticillioides* | YN SJ46 | MK983456 | MK983397 | - |
| *Fusarium vorosii* | NRRL 37605 | KM361647 | KM361665 | - |
| *Fusarium xylarioides* | NRRL 25486 | JX171630 | HM068355 | - |
| *Fusarium xyrophilum* | NRRL 62710 | MN193931 | MN193903 | - |
| *Fusarium xyrophilum* | NRRL 62721 | MN193933 | MN193905 | - |
| *Fusarium xyrophilum* | NRRL 66890 | MN193932 | MN193904 | - |
| *Fusicolla aquaeductuum* | NRRL 20686 | JX171476 | JX171590 | - |
| *Fusicolla sp.* | NRRL 22136 | JX171491 | JX171604 | - |
| *Geejayessia staphyleae* | NRRL 22316 | JX171496 | JX171609 | - |
| *Geejayessia zealandica* | NRRL 22465 | JX171498 | JX171611 | - |
| *Luteonectria albida* | NRRL 22152 | JX171492 | JX171605 | - |
| *Luteonectria nematophila* | NRRL 54600 | JX171552 | JX171664 | - |
| *Macroconia leptosphaeriae* | NRRL 54562 | JX171556 | JX171668 | - |
| *Macroconia sp.* | NRRL 54563 | JX171557 | JX171669 | - |
| *Microcera coccophila* | NRRL 13962 | JX171462 | JX171576 | - |
| *Microcera diploa* | NRRL 36545 | JX171463 | JX171577 | - |
| *Microcera larvarum* | NRRL 20473 | JX171473 | JX171587 | - |
| *Neocosmospora cyanescens* | NRRL 37625 | HM347175 | - | - |
| *Neocosmospora euwallaceae* | NRRL 54724 | JQ038023 | JQ038030 | - |
| *Neocosmospora euwallaceae* | NRRL 54725 | JQ038024 | JQ038031 | - |
| *Neocosmospora euwallaceae* | NRRL 54726 | JQ038025 | JQ038032 | - |
| *Neocosmospora falciformis* | MIW 58 | MN242937 | MN725019 | - |
| *Neocosmospora falciformis* | NRRL 43529 | JX171541 | JX171653 | - |
| *Neocosmospora falciformis* | NRRL 43529 | JX171541 | JX171653 | - |
| *Neocosmospora floridana* | NRRL 62608 | KC691592 | KC691623 | - |
| *Neocosmospora floridana* | NRRL 62628 | KC691593 | KC691624 | - |
| *Neocosmospora floridana* | NRRL 62629 | KC691594 | KC691625 | - |
| *Neocosmospora illudens* | NRRL 22090 | JX171488 | JX171601 | - |
| *Neocosmospora keratoplastica* | ATS92 | MF179712 | KY512536 | - |
| *Neocosmospora keratoplastica* | ATS94 | MF179714 | KY512538 | - |
| *Neocosmospora macrospora* | CPC 28191 | - | LT746331 | LT746218 |
| *Neocosmospora macrospora* | CPC 28192 | - | LT746332 | LT746219 |
| *Neocosmospora macrospora* | CPC 28193 | - | LT746333 | LT746220 |
| *Neocosmospora obliquiseptata* | NRRL 62610 | KC691605 | KC691636 | - |
| *Neocosmospora obliquiseptata* | NRRL 62611 | KC691606 | KC691637 | - |
| *Neocosmospora perseae* | CPC 26829 | - | LT991909 | LT991902 |
| *Neocosmospora perseae* | CPC 26830 | - | LT991910 | LT991903 |
| *Neocosmospora perseae* | CPC 26832 | - | LT991912 | LT991905 |
| *Neocosmospora phaseoli* | CBS 265.50 | KM232226 | KM232375 | HE647964 |
| *Neocosmospora phaseoli* | NRRL 22276 | JX171495 | JX171608 | - |
| *Neocosmospora phaseoli* | NRRL 22411 | KJ511267 | KJ511278 | - |
| *Neocosmospora plagianthi* | NRRL 22632 | JX171501 | JX171614 | - |
| *Neocosmospora pseudensiformis* | NRRL 46517 | KC691615 | KC691645 | - |
| *Neocosmospora solani* | FBF7 | - | MK606410 | MK606409 |
| *Neocosmospora solani* | LEMM_110148 | - | LN828050 | LN827961 |
| *Neocosmospora solani* | LEMM_110266 | - | LN828053 | LN827964 |
| *Neocosmospora solani* | MIM 28 | MN242938 | MN725021 | - |
| *Neocosmospora solani* | MIW 81 | MN242939 | MN724934 | - |
| *Neocosmospora solani* | NRRL 22147 | MG282390 | MG282420 | - |
| *Neocosmospora tonkinense* | CPC 27195 | - | LT746340 | LT746227 |
| *Neocosmospora tuaranensis* | NRRL 22231 | KC691600 | KC691631 | - |
| *Neocosmospora tuaranensis* | NRRL 46518 | KC691601 | KC691632 | - |
| *Neocosmospora tuaranensis* | NRRL 46519 | KC691602 | KC691633 | - |
| *Pycnofusarium rusci* | NRRL 22134 | JX171490 | JX171603 | - |
| *Rectifusarium ventricosum* | NRRL 13953 | JX171461 | JX171575 | - |
| *Rectifusarium ventricosum* | NRRL 20846 | JX171484 | JX171597 | - |
| *Rectifusarium ventricosum* | NRRL 25729 | JX171520 | JX171633 | - |
| *Setofusarium setosum* | NRRL 36526 | JX171539 | JX171651 | - |
